# Supplementary material for: Which companies dominate the packaged food supply of New Zealand and how healthy are their products?
Source: PLoS One. 2021 Jan 26;16(1):e0245225. doi: 10.1371/journal.pone.0245225 (PMC7837499; doi:10.1371/journal.pone.0245225)
Supplement: S1 Table — (DOCX) [file pone.0245225.s001.docx]

# **S1 Table. Food categories excluded from analysis.**

Products from food categories that do not contribute significantly to nutrient intake or are not required to display NIPs were excluded from the analysis. Eggs were excluded as they are a single ingredient, there is no opportunity for reformulation and because of the homogeneity of the nutrient content of eggs from different manufacturers.

| **Food category** | **Number of products** |
| --- | --- |
| Baby foods | 257 |
| Chewing gum | 38 |
| Cough lollies | 10 |
| Dietary supplements | 22 |
| Herbs and spices | 182 |
| Plain tea and coffee | 100 |
| Products not able to be categorised (e.g., gelatine, raising agents, arrowroot) | 24 |
| Eggs | 90 |
| **Total** | **723** |
