# Supplementary material for: Which companies dominate the packaged food supply of New Zealand and how healthy are their products?
Source: PLoS One. 2021 Jan 26;16(1):e0245225. doi: 10.1371/journal.pone.0245225 (PMC7837499; doi:10.1371/journal.pone.0245225)
Supplement: S2 Table — (DOCX) [file pone.0245225.s002.docx]

# **S2 Table. Classifying foods in Nutritrack according to the level of processing (NOVA).**

There are four categories of degree of processing. The categories are based on the NOVA system of categorisation^(1)^. Categories

- Natural and minimally processed (MP)
- Culinary ingredients (PI)
- Processed (P)
- Ultra-processed (UP)

Items are assigned based on their level and type of processing, the difference in the item to the original form and the type and function of added ingredients. Firstly, assign a level of processing to a Nutritrack sub-category according to the detailed description of categories outlined in: The Food System: NOVA The star shines bright. World Nutrition 2016 7:#1-3, Jan-March 2016^(1)^. Use Table 1 and Table 2 to guide decision-making.

If 70% of products are of the same degree of processing (e.g. all ultra-processed) then assign the category that degree of processing. **Except**, if the category ranges from minimally processed and ultra-processed assign at an individual level, unless there is an outlier. For example, plain yoghurt includes MP, P and UP products so is categorised at an individual level. Frozen fruit has 66 MP products, 1 UP and 1 P so category assigned as minimally processed.

**Table 1: Classifying level of processing by ingredients**

| **Type of Ingredient** | **Decision** | **Category** |
| --- | --- | --- |
| Ingredients in a product | If all MP  If mix of MP and P ingredients  If any ingredients are UP | MP  P  UP |
| Preservative | If preservative to preserve properties of original food but no other additives | MP or P |
| Colour | Natural colour in hard Cheese  Any other added colour including natural | P  UP |
| Flavour | Any added flavour including ‘natural flavour’  Except: ingredients added for flavouring that are MP e.g. lemon juice, thyme, natural almond essence, natural vanilla essence and no UP ingredients | UP  P |
| Glaze | If only sugar and water and no other additives | P |
| Artificial sweetener | If contains artificial sweetener | UP |
| Stabilisers | If essential to integrity of product and no UP ingredients  If not essential to integrity of product | P  UP |
| Anti-caking | If only additive | P |
| Carbonation | If naturally carbonated at water source  If only additive to sparkling water | MP  P |
| Propellants | If only additive | P |
| Firming agents | If essential for function (e.g. tofu, soft cheeses)  If not essential or includes other additives | P  UP |
| Industrial processes | If plain dried pasta and noodles.  If extruded, moulded, pre- processing for frying | P  UP |
| Fortification | If voluntary or mandatory fortification (iodine, folate, calcium in dairy substitutes) and not considered UP by any other rule  If juice with Vitamin C added as preservative and no other added ingredients  If replacing nutrients lost in processing and not considered UP by any other rule  If other added vitamins and minerals not acting as preservative or mandatory fortification | MP or P  MP  MP or P  UP |
| Starches | If a modified starch  If tapioca starch, cornflour or pea starch added as the only additive to MP or P foods | UP  P |
| Other additives | Items that contain bulking, anti-bulking, de-foaming, emulsifiers, sequestrants, humectants, casein, lactose, whey, gluten, hydrogenated oils, hydrolysed proteins, soy protein isolate, maltodextrin, invert sugar, high fructose corn syrup | UP |
| Added sugar, oil, salt | If no added sugar, oil or salt  If added sugar, oil or salt  If other additives, then classify according to these | MP  P |
| Probiotics | If only additive to MP ingredient (e.g. plain yoghurt) | MP |
| Hydrogenated fats | If contains hydrogenated fats (e.g. margarine, table spreads) | UP |
| Ultra-heat treatment | I no other additives (e.g. UHT milk) | MP |

MP, Minimally processed

P, Processed

UP, Ultra-processed

**Table 2: Decisions for selected food categories**

| **Food category** | **Decision** | **Category** |
| --- | --- | --- |
| Dried fruit powders | If only dried fruit powder only | MP |
| Dried pasta and dried plain noodles | If flour, water, MP ingredients only, can be extruded shapes  If contains salt and/or citric acid  If contains other additives | MP  P  UP |
| Fresh plain pasta and fresh plain poodles | If contains no salt or additives  If contains salt but no other additives  If contains other additives than determined by rule for the additive | MP  P |
| Chilled soups | If a combination of MP and culinary ingredients and may have added salt, sugar, yeast extract, corn starch, herbs and spices  If contains other additives, e.g. maize thickener, food acid, flavour enhancer | P  UP |
| Other flavoured soft cheese | If MP foods or culinary ingredients added to cheese or milk and ingredients necessary to cheese-making (e.g. culture, rennet)  If contains other additives, e.g. thickeners, cultured dextrose, flavour, culture, stabiliser | P  UP |
| Natural yoghurt  Lactose-free yoghurt | If contains only milk, cream, milk solids, cultures, probiotics  If contains only one additional additive with a function related to yoghurt, e.g. stabilizer, thickener, pectin, gelatine. May also contain one added sugar ingredient  If contain more than one additional additive with a function related to yoghurt and/or other additives e.g. flavour, colour | MP  P  UP |
| Fruit bars | If only fruit puree or fruit juice concentrate  If added culinary ingredient, eg oil  If other additives, eg fibre, gelling agents, flavours, colours, acids | MP  P  UP |
| Dried fruit | If oil added to stop fruit sticking together, e.g. raisins  If sulphite added as a necessary preservative | MP  MP |
| Pancetta and prosciutto | If only pork & salt & preservative  If other additives, e.g. dextrose, lactose, antioxidant, flavourings | P  UP |
| Dried meat | If only meat, salt, spices, vinegar  If other additives, e.g. gum, maltodextrin, flavour, acidity regulator, modified protein | P  UP |
| Juice | If juice or reconstituted juice with vitamin C and/or citric acid  If added sugar or salt (tomato juice) and one or two of the following: preservative or stabiliser or Vitamin C  If added flavour (even ‘natural’ flavour) or 3+ additives, with or without added sugar or salt. | MP  P  UP |
| Tomato-based pasta sauce | If contains tomatoes, salt, sugar, 1 food acid, other vegetables, cornflour, herbs  If also contains thickener, modified starch, dextrose, yeast extract, firming agent | P  UP |

Culinary ingredients: oil, butter, sugar, salt
